# 2-Amino-4-methyl­pyridinium (*E*)-3-carb­oxy­prop-2-enoate

**DOI:** 10.1107/S1600536810026292

**Published:** 2010-07-10

**Authors:** Madhukar Hemamalini, Hoong-Kun Fun

**Affiliations:** a X-ray Crystallography Unit, School of Physics, Universiti Sains Malaysia, 11800 USM, Penang, Malaysia

## Abstract

In the title salt, C_6_H_9_N_2_^+^·C_4_H_3_O_4_^−^, the dihedral angle between the pyridine ring and the plane formed by the hydrogen fumarate anion is 85.67 (6)°. Excluding the amino and methyl groups, the atoms of the cation are coplanar, with a maximum deviation of 0.005 (1) Å. In the crystal structure, the protonated N atom and the 2-amino group of the cation are hydrogen bonded to the carboxyl­ate O atoms of the anion *via* a pair of N—H⋯O hydrogen bonds, forming an *R*_2_^2^(8) ring motif. These motifs are further connected through N—H⋯O and C—H⋯O hydrogen bonds, leading to a supra­molecular chain along the *c* axis. These chains are further cross-linked *via* a pair of O—H⋯O hydrogen bonds involving centrosymmetrically related hydrogen fumarate anions, forming a two-dimensional network parallel to (101). These planes are further interconnected by O—H⋯O interactions into a three-dimensional network.

## Related literature

For applications of inter­molecular inter­actions, see: Lam & Mak (2000 ▶). For related structures, see: Büyükgüngör & Odabąsoğlu (2006 ▶); Hosomi *et al.* (2000 ▶); Smith *et al.* (2007 ▶); Cao *et al.* (2004 ▶); Natarajan *et al.* (2009 ▶). For hydrogen-bond motifs, see: Bernstein *et al.* (1995 ▶). For reference bond-length data, see: Allen *et al.* (1987 ▶). For the stability of the temperature controller used in the data collection, see: Cosier & Glazer (1986 ▶).
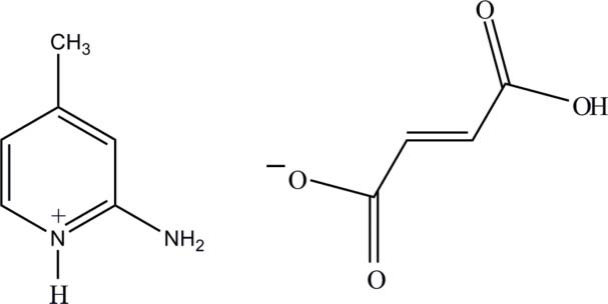


## Experimental

### 

#### Crystal data

C_6_H_9_N_2_^+^·C_4_H_3_O_4_^−^
*M* *_r_* = 224.22
Monoclinic, 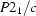

*a* = 5.0058 (16) Å
*b* = 19.814 (7) Å
*c* = 11.286 (4) Å
β = 108.332 (13)°
*V* = 1062.6 (6) Å^3^
*Z* = 4
Mo *K*α radiation
μ = 0.11 mm^−1^
*T* = 100 K
0.36 × 0.10 × 0.07 mm

#### Data collection

Bruker APEXII DUO CCD area-detector diffractometer
Absorption correction: multi-scan (*SADABS*; Bruker, 2009 ▶) *T*_min_ = 0.962, *T*_max_ = 0.992
12900 measured reflections
3387 independent reflections
2573 reflections with *I* > 2σ(*I*)
*R*_int_ = 0.041

#### Refinement

*R*[*F* ^2^ > 2σ(*F* ^2^)] = 0.046
*wR*(*F* ^2^) = 0.168
*S* = 1.07
3387 reflections
181 parameters
All H-atom parameters refined
Δρ_max_ = 0.51 e Å^−3^
Δρ_min_ = −0.32 e Å^−3^

### 

Data collection: *APEX2* (Bruker, 2009 ▶); cell refinement: *SAINT* (Bruker, 2009 ▶); data reduction: *SAINT*; program(s) used to solve structure: *SHELXTL* (Sheldrick, 2008 ▶); program(s) used to refine structure: *SHELXTL*; molecular graphics: *SHELXTL*; software used to prepare material for publication: *SHELXTL* and *PLATON* (Spek, 2009 ▶).

## Supplementary Material

Crystal structure: contains datablocks global, I. DOI: 10.1107/S1600536810026292/wn2399sup1.cif

Structure factors: contains datablocks I. DOI: 10.1107/S1600536810026292/wn2399Isup2.hkl

Additional supplementary materials:  crystallographic information; 3D view; checkCIF report

## Figures and Tables

**Table 1:** Hydrogen-bond geometry (Å, °)

*D*—H⋯*A*	*D*—H	H⋯*A*	*D*⋯*A*	*D*—H⋯*A*
O4—H1*O*4⋯O3^i^	0.928 (19)	1.720 (19)	2.6472 (17)	179 (2)
N1—H1*N*1⋯O1^ii^	0.987 (18)	1.689 (18)	2.6761 (16)	179.5 (17)
N2—H1*N*2⋯O2^ii^	0.994 (18)	1.804 (18)	2.7979 (16)	179.0 (16)
N2—H2*N*2⋯O1^iii^	0.816 (19)	2.028 (19)	2.8320 (18)	168.5 (18)
C2—H2*A*⋯O3^iv^	0.964 (18)	2.596 (18)	3.349 (2)	135.1 (14)
C5—H5*A*⋯O2^v^	1.015 (16)	2.239 (16)	3.189 (2)	155.0 (13)
C6—H6*B*⋯O3^iv^	0.954 (19)	2.592 (19)	3.360 (2)	137.8 (16)

Symmetry codes: (i) 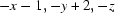; (ii) 
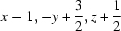
; (iii) 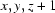; (iv) 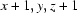; (v) 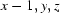.

**Fig. 1.**
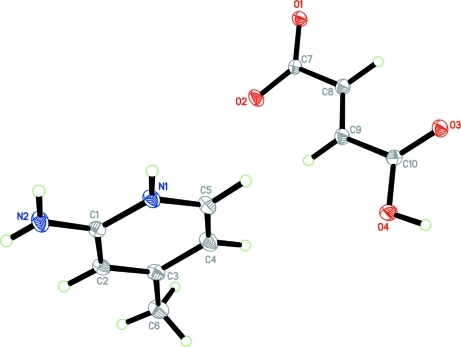
The asymmetric unit of the title compound. Displacement ellipsoids are drawn at the 50% probability level. Hydrogen atoms are shown as spheres of arbitrary radius.

**Fig. 2.**
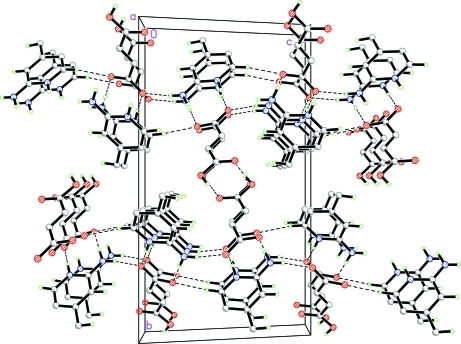
The crystal packing of the title compound, showing hydrogen-bonded (dashed lines) 2D networks parallel to (010). H atoms not involved in the intermolecular interactions have been omitted for clarity.

## supplementary crystallographic information

### Comment

Intermolecular interactions are responsible for crystal packing and gaining an understanding of them allows us to comprehend collective properties and permits the design of new crystals with specific physical and chemical properties (Lam & Mak, 2000). Fumaric acid, a key intermediate in organic acid biosynthesis, is known to readily form adducts/complexes with other organic molecules. The crystal structures of 2,6-diaminopyridinium hydrogen fumarate (Büyükgüngör & Odabąsoǧlu, 2006), triethylammonium hydrogen fumarate (Hosomi *et al.*, 2000), anhydrous guanidinium hydrogen fumarate (Smith *et al.*, 2007), tiamulin hydrogen fumarate methanol (Cao *et al.*, 2004) and glycinium hydrogen fumarate glycine solvate monohydrate (Natarajan *et al.*, 2009) have been reported. The present study has been undertaken to study the hydrogen bonding patterns involving the hydrogen fumarate anion with the 2-amino-4-methylpyridinium cation.

The asymmetric unit of the title compound consists of a 2-amino-4-methyl pyridinium cation and a hydrogen fumarate anion (Fig. 1). In the 2-amino- 4-methylpyridinium cation, a wider than normal angle [C1—N1—C5 121.94 (11)°] is subtended at the protonated N1 atom.  The C10—O3 bond distance of 1.2259 (16) Å is much shorter than the C10—O4 bond distance of 1.3224 (15) Å, suggesting that the carboxyl group is not deprotonated in the crystal structure. The dihedral angle between the pyridine ring and the plane formed by the hydrogen fumarate anion is 85.67 (6)°. Excluding amino and methyl groups, the atoms of the cation are coplanar, with a maximum deviation of 0.005 (1) Å for atom C2. The bond lengths (Allen *et al.*, 1987) and angles are normal.

In the crystal packing (Fig. 2), the protonated N1 atom and the 2-amino group (N2) are hydrogen-bonded to the carboxylate oxygen atoms (O1 and O2) via a pair of N—H···O hydrogen bonds, forming a *R*_2_^2^(8) ring motif (Bernstein *et al.*, 1995). Furthermore, these motifs are connected through N—H···O and C—H···O hydrogen bonds (Table 1), leading to a one-dimensional supramolecular chain along the *c*-axis. These chains are further connected via a pair of O—H···O hydrogen bonds involving centrosymmetric hydrogen fumarate anions, forming a two-dimensional network parallel to (010). These planes are further interconnected by O4–H1O4···O3 hydrogen bonds into a 3D network.

### Experimental

A hot methanol solution (20 ml) of 2-amino-4-methylpyridine (54 mg, Aldrich) and fumaric acid (58 mg, Merck) were mixed and warmed over a heating magnetic stirrer hotplate for a few minutes. The resulting solution was allowed to cool slowly at room temperature and crystals of the title compound appeared after a few days.

### Refinement

All the H atoms were located from a difference Fourier map and refined freely [C—H = 0.953 (19)–1.027 (17) Å; N—H = 0.816 (19)–0.994 (18) Å and O—H = 0.93 (2) Å].

### Crystal data

**Table:**

C_6_H_9_N_2_^+^·C_4_H_3_O_4_^−^	*F*(000) = 472
*M**_r_* = 224.22	*D*_x_ = 1.402 Mg m^−^^3^
Monoclinic, *P*2_1_/*c*	Mo *K*α radiation, λ = 0.71073 Å
Hall symbol: -P 2ybc	Cell parameters from 2803 reflections
*a* = 5.0058 (16) Å	θ = 2.8–31.0°
*b* = 19.814 (7) Å	µ = 0.11 mm^−^^1^
*c* = 11.286 (4) Å	*T* = 100 K
β = 108.332 (13)°	Needle, colourless
*V* = 1062.6 (6) Å^3^	0.36 × 0.10 × 0.07 mm
*Z* = 4	

### Data collection

**Table:**

Bruker APEXII DUO CCD area-detector diffractometer	3387 independent reflections
Radiation source: fine-focus sealed tube	2573 reflections with *I* > 2σ(*I*)
graphite	*R*_int_ = 0.041
φ and ω scans	θ_max_ = 31.1°, θ_min_ = 2.1°
Absorption correction: multi-scan (*SADABS*; Bruker, 2009)	*h* = −7→7
*T*_min_ = 0.962, *T*_max_ = 0.992	*k* = −22→28
12900 measured reflections	*l* = −16→16

### Refinement

**Table:**

Refinement on *F*^2^	Primary atom site location: structure-invariant direct methods
Least-squares matrix: full	Secondary atom site location: difference Fourier map
*R*[*F*^2^ > 2σ(*F*^2^)] = 0.046	Hydrogen site location: inferred from neighbouring sites
*wR*(*F*^2^) = 0.168	All H-atom parameters refined
*S* = 1.07	*w* = 1/[σ^2^(*F*_o_^2^) + (0.106*P*)^2^ + 0.0446*P*] where *P* = (*F*_o_^2^ + 2*F*_c_^2^)/3
3387 reflections	(Δ/σ)_max_ = 0.001
181 parameters	Δρ_max_ = 0.51 e Å^−^^3^
0 restraints	Δρ_min_ = −0.32 e Å^−^^3^

### Special details

**Table:**

Experimental. The crystal was placed in the cold stream of an Oxford Cryosystems Cobra open-flow nitrogen cryostat (Cosier & Glazer, 1986) operating at 100.0 (1) K.
Geometry. All s.u.'s (except the s.u. in the dihedral angle between two l.s. planes) are estimated using the full covariance matrix. The cell s.u.'s are taken into account individually in the estimation of s.u.'s in distances, angles and torsion angles; correlations between s.u.'s in cell parameters are only used when they are defined by crystal symmetry. An approximate (isotropic) treatment of cell s.u.'s is used for estimating s.u.'s involving l.s. planes.
Refinement. Refinement of F^2^ against ALL reflections. The weighted R-factor wR and goodness of fit S are based on F^2^, conventional R-factors R are based on F, with F set to zero for negative F^2^. The threshold expression of F^2^ > 2σ(F^2^) is used only for calculating R-factors(gt) etc. and is not relevant to the choice of reflections for refinement. R-factors based on F^2^ are statistically about twice as large as those based on F, and R- factors based on ALL data will be even larger.

### Fractional atomic coordinates and isotropic or equivalent isotropic displacement parameters (Å^2^)

**Table:**

	*x*	*y*	*z*	*U*_iso_*/*U*_eq_	
N1	−0.1142 (2)	0.80864 (6)	0.55566 (10)	0.0138 (2)	
N2	0.0376 (2)	0.76324 (6)	0.75413 (11)	0.0166 (2)	
C1	0.0731 (3)	0.80768 (7)	0.67234 (11)	0.0129 (3)	
C2	0.2978 (3)	0.85492 (7)	0.70188 (12)	0.0143 (3)	
C3	0.3220 (3)	0.90042 (7)	0.61461 (12)	0.0153 (3)	
C4	0.1210 (3)	0.89903 (7)	0.49325 (12)	0.0176 (3)	
C5	−0.0911 (3)	0.85296 (7)	0.46726 (12)	0.0159 (3)	
C6	0.5539 (3)	0.95182 (8)	0.64658 (13)	0.0198 (3)	
O1	0.4413 (2)	0.77430 (5)	−0.00598 (9)	0.0167 (2)	
O2	0.5841 (2)	0.82660 (5)	0.17910 (9)	0.0187 (2)	
O3	−0.33328 (19)	0.94128 (5)	−0.04434 (9)	0.0170 (2)	
O4	−0.2087 (2)	0.98609 (5)	0.14785 (9)	0.0184 (2)	
C7	0.4153 (3)	0.81746 (7)	0.07303 (11)	0.0129 (3)	
C8	0.1559 (3)	0.86036 (7)	0.03171 (12)	0.0144 (3)	
C9	0.0841 (3)	0.90251 (7)	0.10791 (12)	0.0150 (3)	
C10	−0.1721 (3)	0.94438 (7)	0.06278 (12)	0.0132 (3)	
H1O4	−0.368 (4)	1.0119 (9)	0.1114 (16)	0.020*	
H1N1	−0.278 (4)	0.7781 (9)	0.5333 (16)	0.016*	
H1N2	−0.124 (4)	0.7315 (9)	0.7264 (16)	0.016*	
H2N2	0.141 (4)	0.7622 (9)	0.8262 (17)	0.016*	
H2A	0.428 (4)	0.8529 (9)	0.7855 (16)	0.016*	
H4A	0.137 (4)	0.9308 (9)	0.4299 (16)	0.016*	
H5A	−0.244 (3)	0.8495 (9)	0.3834 (15)	0.016*	
H6A	0.655 (4)	0.9509 (10)	0.5852 (16)	0.020*	
H6B	0.683 (4)	0.9461 (9)	0.7285 (17)	0.020*	
H6C	0.457 (4)	0.9960 (10)	0.6385 (16)	0.020*	
H8A	0.029 (4)	0.8563 (9)	−0.0593 (16)	0.016*	
H9A	0.198 (4)	0.9063 (9)	0.1949 (16)	0.016*	

### Atomic displacement parameters (Å^2^)

**Table:**

	*U*^11^	*U*^22^	*U*^33^	*U*^12^	*U*^13^	*U*^23^
N1	0.0130 (5)	0.0153 (6)	0.0118 (5)	−0.0028 (4)	0.0020 (4)	−0.0010 (4)
N2	0.0164 (5)	0.0181 (6)	0.0123 (5)	−0.0050 (4)	0.0004 (4)	0.0012 (4)
C1	0.0126 (5)	0.0137 (6)	0.0119 (5)	−0.0010 (4)	0.0031 (4)	−0.0015 (4)
C2	0.0145 (5)	0.0156 (6)	0.0127 (5)	−0.0027 (4)	0.0039 (4)	−0.0025 (5)
C3	0.0161 (6)	0.0164 (6)	0.0146 (6)	−0.0032 (5)	0.0064 (5)	−0.0024 (5)
C4	0.0206 (6)	0.0197 (7)	0.0135 (6)	−0.0038 (5)	0.0065 (5)	−0.0001 (5)
C5	0.0167 (6)	0.0184 (7)	0.0115 (5)	−0.0012 (5)	0.0029 (4)	−0.0011 (5)
C6	0.0208 (6)	0.0206 (7)	0.0188 (6)	−0.0082 (5)	0.0074 (5)	−0.0024 (5)
O1	0.0160 (4)	0.0174 (5)	0.0143 (4)	0.0045 (3)	0.0013 (3)	−0.0027 (4)
O2	0.0155 (4)	0.0238 (6)	0.0132 (4)	0.0066 (4)	−0.0007 (3)	−0.0031 (4)
O3	0.0152 (4)	0.0195 (5)	0.0144 (4)	0.0052 (3)	0.0019 (4)	−0.0021 (4)
O4	0.0167 (5)	0.0194 (5)	0.0169 (5)	0.0057 (4)	0.0022 (4)	−0.0046 (4)
C7	0.0121 (5)	0.0129 (6)	0.0131 (5)	0.0014 (4)	0.0033 (4)	0.0014 (4)
C8	0.0127 (5)	0.0150 (6)	0.0147 (6)	0.0027 (4)	0.0030 (4)	−0.0002 (5)
C9	0.0131 (5)	0.0166 (6)	0.0141 (6)	0.0028 (4)	0.0023 (4)	−0.0003 (5)
C10	0.0121 (5)	0.0134 (6)	0.0143 (6)	−0.0001 (4)	0.0043 (4)	−0.0009 (4)

### Geometric parameters (Å, °)

**Table:**

N1—C1	1.3553 (16)	C6—H6A	0.977 (18)
N1—C5	1.3611 (17)	C6—H6B	0.953 (19)
N1—H1N1	0.987 (18)	C6—H6C	0.991 (19)
N2—C1	1.3276 (17)	O1—C7	1.2714 (15)
N2—H1N2	0.994 (18)	O2—C7	1.2424 (16)
N2—H2N2	0.816 (19)	O3—C10	1.2259 (16)
C1—C2	1.4202 (18)	O4—C10	1.3224 (15)
C2—C3	1.3683 (18)	O4—H1O4	0.93 (2)
C2—H2A	0.964 (17)	C7—C8	1.4987 (18)
C3—C4	1.4219 (19)	C8—C9	1.3269 (18)
C3—C6	1.5006 (19)	C8—H8A	1.027 (17)
C4—C5	1.3604 (19)	C9—C10	1.4769 (18)
C4—H4A	0.976 (17)	C9—H9A	0.970 (18)
C5—H5A	1.015 (17)		
			
C1—N1—C5	121.94 (11)	N1—C5—H5A	115.3 (10)
C1—N1—H1N1	120.4 (10)	C3—C6—H6A	110.5 (11)
C5—N1—H1N1	117.6 (10)	C3—C6—H6B	112.9 (11)
C1—N2—H1N2	118.4 (10)	H6A—C6—H6B	110.0 (16)
C1—N2—H2N2	121.9 (13)	C3—C6—H6C	105.0 (11)
H1N2—N2—H2N2	119.7 (16)	H6A—C6—H6C	107.3 (15)
N2—C1—N1	118.80 (11)	H6B—C6—H6C	110.9 (15)
N2—C1—C2	122.92 (12)	C10—O4—H1O4	108.6 (11)
N1—C1—C2	118.27 (11)	O2—C7—O1	125.82 (12)
C3—C2—C1	120.53 (12)	O2—C7—C8	118.51 (11)
C3—C2—H2A	123.2 (11)	O1—C7—C8	115.67 (11)
C1—C2—H2A	116.3 (11)	C9—C8—C7	122.70 (12)
C2—C3—C4	119.00 (12)	C9—C8—H8A	119.2 (10)
C2—C3—C6	120.72 (12)	C7—C8—H8A	118.1 (10)
C4—C3—C6	120.28 (12)	C8—C9—C10	120.85 (12)
C5—C4—C3	119.24 (12)	C8—C9—H9A	120.8 (11)
C5—C4—H4A	121.0 (10)	C10—C9—H9A	118.3 (11)
C3—C4—H4A	119.7 (11)	O3—C10—O4	123.28 (12)
C4—C5—N1	121.02 (12)	O3—C10—C9	122.87 (11)
C4—C5—H5A	123.7 (10)	O4—C10—C9	113.83 (11)
			
C5—N1—C1—N2	179.79 (12)	C3—C4—C5—N1	0.3 (2)
C5—N1—C1—C2	0.38 (18)	C1—N1—C5—C4	−0.8 (2)
N2—C1—C2—C3	−178.88 (12)	O2—C7—C8—C9	−7.3 (2)
N1—C1—C2—C3	0.51 (19)	O1—C7—C8—C9	172.67 (13)
C1—C2—C3—C4	−1.0 (2)	C7—C8—C9—C10	179.34 (11)
C1—C2—C3—C6	178.11 (12)	C8—C9—C10—O3	2.0 (2)
C2—C3—C4—C5	0.5 (2)	C8—C9—C10—O4	−177.01 (12)
C6—C3—C4—C5	−178.53 (13)		

### Hydrogen-bond geometry (Å, °)

**Table:**

*D*—H···*A*	*D*—H	H···*A*	*D*···*A*	*D*—H···*A*
O4—H1O4···O3^i^	0.928 (19)	1.720 (19)	2.6472 (17)	179 (2)
N1—H1N1···O1^ii^	0.987 (18)	1.689 (18)	2.6761 (16)	179.5 (17)
N2—H1N2···O2^ii^	0.994 (18)	1.804 (18)	2.7979 (16)	179.0 (16)
N2—H2N2···O1^iii^	0.816 (19)	2.028 (19)	2.8320 (18)	168.5 (18)
C2—H2A···O3^iv^	0.964 (18)	2.596 (18)	3.349 (2)	135.1 (14)
C5—H5A···O2^v^	1.015 (16)	2.239 (16)	3.189 (2)	155.0 (13)
C6—H6B···O3^iv^	0.954 (19)	2.592 (19)	3.360 (2)	137.8 (16)

Symmetry codes: (i) −*x*−1, −*y*+2, −*z*; (ii) *x*−1, −*y*+3/2, *z*+1/2; (iii) *x*, *y*, *z*+1; (iv) *x*+1, *y*, *z*+1; (v) *x*−1, *y*, *z*.
